# Association between post-stroke depressiveness and the utilization of healthcare services three months after the stroke

**DOI:** 10.1038/s41598-025-12875-x

**Published:** 2025-08-02

**Authors:** Raphaela Artner, Christine Meisinger, Michael Ertl, Markus Naumann, Jakob Linseisen, Timo Schmitz

**Affiliations:** 1 Institute for Medical Information Processing, Biometry, and Epidemiology-IBE, LMU Munich, Munich, Germany; 2 Pettenkofer School of Public Health, Munich, Germany; 3 Epidemiology, Medical Faculty, University of Augsburg, Augsburg, Germany; 4 Department of Neurology and Clinical Neurophysiology, University Hospital Augsburg, Augsburg, Germany

**Keywords:** Stroke, Depressiveness, Healthcare utilization, Post-stroke depression, Cohort study, Stroke, Epidemiology, Depression

## Abstract

Post-stroke depression affects approximately one-third of stroke survivors and can result in various adverse outcomes. With the rising prevalence of strokes, understanding the impact of post-stroke depression on healthcare utilization is crucial. The study examined the association between patient-reported post-stroke depressiveness and the healthcare service utilization three months post-stroke. The prospective Stroke Cohort Augsburg (SCHANA) study assessed post-stroke depressiveness using the Patient Health Questionnaire (PHQ-9) during hospital stay. Participants reported their utilization of medical and therapeutic services, rehabilitation, and inpatient hospital stays three months post-discharge. Multivariable adjusted linear and binary logistic regression analyses examined the association between post-stroke depressiveness and healthcare service utilization. Among 546 participants included into the analyses, 57.1% had no depression, 28.8% had mild depression and 14.9% had moderate to severe depression. Higher PHQ-9 scores were associated with a significantly increased likelihood of inpatient hospital treatment (OR = 1.07; 95% CI: 1.01–1.14; *p* = 0.016), attendance at rehabilitation programs (OR = 1.09; 95% CI: 1.03–1.15; *p* = 0.003), and more frequent utilization of general practitioners/internists (beta = 0.10; 95% CI: 0.01–0.19; *p* = 0.036). In conclusion, post-stroke depressiveness was associated with increased healthcare service utilization. Further studies are required to gain a more comprehensive understanding.

**Supplementary Information:**

The online version contains supplementary material available at 10.1038/s41598-025-12875-x.

## Introduction

Stroke continues to represent a substantial global health burden, accounting for a considerable proportion of disability and morbidity among adults globally and in Germany^1–3^. The economic burden of stroke in Germany is placed somewhere in the middle range compared to other industrialized countries^4^. However, an evaluation of a German stroke database in the year 2002 shows that the costs for each stroke event are enormous^5^. Notwithstanding the progress made in the treatment and prevention of stroke, the economic burden will persist, largely due to the anticipated rise in the incidence of stroke in the future^1,3,6^. Stroke can result in several long-term impairments, including but not limited to mobility issues, cognitive deficits, communication difficulties, and sensory changes^7^. Among these, post-stroke depression (PSD) emerges as a common mental health issue, affecting approximately one-third of stroke survivors^8,9^. The consequences of PSD are far-reaching, including suboptimal recovery, reduced quality of life, increased risk of recurrent stroke events, and increased mortality rates^9–13^.

A longitudinal analysis conducted in the United States revealed that two-thirds of patients who were screened positive for PSD did not receive depression treatment^14^. Moreover, the cost of hospital treatment for stroke patients with comorbid depression is greater than for patients with stroke alone^15^. In light of these challenges regarding the economic burden, the rising prevalence of stroke, and the severe outcomes of PSD, it is imperative to assess the quality of post-stroke care. A particular focus should be placed on understanding the impact of PSD on healthcare utilization.

The objective of this study is to determine the association between symptoms of depression and the utilization of healthcare services among adult patients after stroke. To this end, data from the Stroke Cohort Augsburg (SCHANA) study was used to analyze the association between patient-reported depressive symptoms and utilization of healthcare services (doctor visits, inpatient hospital stays, outpatient or inpatient rehabilitation stays, and utilization of therapy services at 3 months post-stroke).

## Materials and methods

### Study design

The present analysis was based on data from the SCHANA study. This observational single-center study included all patients aged 18 years and older who were treated at the University Hospital Augsburg for an incident or recurrent ischemic or hemorrhagic stroke between September 2018 and November 2019. The aim of this study was to gain information about risk factors, diagnostic and treatment procedures, and the long-term disease course^16^. Therefore, both clinical parameters and self-reported data (interview, questionnaire) were recorded. In total, a baseline survey and 2 follow-up surveys were conducted for each patient. The baseline examination took place during the hospital stay, follow-up surveys were performed 3 months and 12 months after hospital discharge. For additional information regarding this study, refer to the published study protocol^16^.

Data of 945 participants included into the SCHANA study were available for the present analysis. During the time of the recruitment, approximately 2600 stroke patients were treated at the university hospital Augsburg. Main causes for non-participation were discharge before contact was made (around 50% of all non-participants), non-participation due to aphasia, cognitive impairment, palliative care, language barrier etc. (25%), around 17% of patients declined participation and 7% deceased or did not participate due to other reasons. Non-participation due to a language barrier usually meant that the patient was unable to understand the consent form and accordingly was not able to give informed consent.

**Fig. 1:**
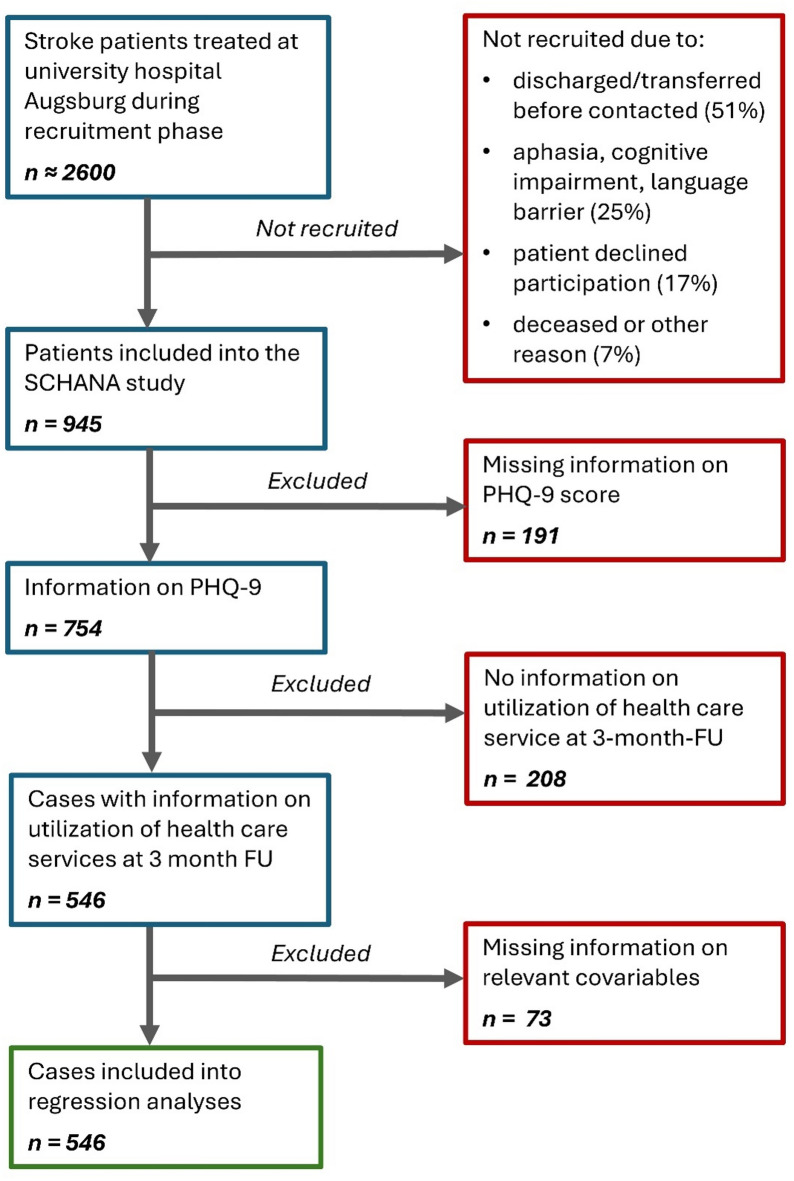
The flow chart displays all inclusions and exclusions and how the final sample size.

Of the 945 patients that were included, 9 patients died during their hospital stay and 182 participants (19.4%) did not complete the PHQ-9 questionnaire. Of the remaining 754 participants, there was no 3-month follow-up for 208 patients, leaving 546 patients that were included into this analysis. Figure 1 displays all inclusion and exclusion in the form of a flow chart.

Written informed consent was obtained from all participants or legal caregivers. The study was conducted according to the Declaration of Helsinki. Ethical approval was granted by the Ethics Committee of the Ludwig-Maximilians-Universität München (Reference-Number: 18–196) in August 2018.

### Data collection

After inclusion in the study the participants were requested to complete a standardized questionnaire and to undergo an interview with a trained study nurse. The study nurses were trained according to a standard operating procedure (SOP). The SOP describes the exact procedure of the patient recruitment, the interviews and the data collection. After extensive training and practice in conducting interviews, several interviews were conducted under the supervision of the principal investigator. The study nurses were only deployed for patient recruitment and interviews once they had successfully passed a certification test.

If a patient suffered from dementia or another disease that significantly impaired cognitive abilities, written informed consent was obtained from the responsible legal caregivers. In such cases, a proxy interview was conducted. Patients who were unable to comprehend the study information due to language barriers were excluded from the study. Patients with a post-stroke cognitive impairment were not excluded from the study as a whole; however, they were excluded from analyses based exclusively on questionnaire data provided by the patient. Cognitive impairment was defined as a score of less than 27 in a mini-mental state test^16^.

Three and 12 months after discharge from the hospital, patients received postal questionnaires and if necessary, also telephone interviews. If the baseline questionnaire has been completed by a legal caregiver, the follow-up questionnaires were also directed to the caregiver. The present analysis used data from the baseline assessment and the 3-month follow-up survey.

### Survey data

Various standardized questionnaires were used for the baseline and the 3-month follow-up survey^13^. The relevant questionnaires for the present study are presented below.

#### Depressiveness assessment

Depressive symptoms were assessed using the standardized 9-item Patient Health Questionnaire-9 (PHQ-9) at the baseline survey during the inpatient hospital treatment. The PHQ-9 is translated and validated in multiple languages and widely used as a patient-reported questionnaire for assessing symptoms of depression^17–19^. Each of the 9 Diagnostic and Statistical Manual of Mental Disorders (DSM-IV) criteria is represented by one question that the participant rates on a Likert scale from 0 (not at all) to 3 (nearly every day). The single scores are then added up, ranging from 0 to 27 with higher scores indicating a more severe level of depression^20^. A total score below 5 can be interpreted as the absence of depression. A score of 5 to 9 indicates a mild depression. A value of 10 and above indicates a moderate to severe depression^21^. It must be mentioned, that the PHQ-9 only evaluates symptoms of depression based on patient self-reported data, which must be clearly distinguished from a clinically confirmed diagnosis of depression. All patients were required to fill out the PHQ-9 form by themselves, there were no proxy interviews for this questionnaire, which also rules out patients with cognitive impairment for the present analysis. The results of the PHQ-9 questionnaire were not reviewed by the health care personnel and consequently it can be ruled out, that the questionnaire has acted as an intervention itself.

#### Assessment of utilization of healthcare services

Utilization data were assessed at the 3-month follow-up survey. The survey included yes/no questions regarding the utilization as well as the number of visits of various medical disciplines, therapies, inpatient stays in hospitals and rehabilitation during the last 3 months since the discharge from the hospital. In particular, the utilization of medical services included the following disciplines: general practitioner (GP), internist, neurologist, urologist, surgeon, ophthalmologist, psychiatrist or psychotherapist and other physicians. In Germany, internal medicine doctors often take on the role of the family doctor/general practitioner, so we decided to combine GP and internist into one variable “GP and/or internist”. As the number of patients that visited a specific physician other than GP/internist at 3-month follow-up was quite small, all other medical disciplines were grouped together as “other physicians”.

The category of therapy services included questions on the utilization of various forms of therapy services, including occupational therapy, speech therapy, physiotherapy, chiropractic therapy, alternative practitioner, acupuncture, homeopathy, osteopathy and other forms of therapy services. In the present study occupational therapy, speech therapy and physiotherapy were grouped together as “therapy services”. All other categories were excluded from the analyses as they are self-pay services and are usually not covered by health insurance in Germany.

Furthermore, the participants were queried in the follow-up survey to provide information if they had an inpatient treatment in the hospital and an inpatient or outpatient rehabilitation over the past 3 months. In addition to the dichotomous questions regarding the utilization of various healthcare services, the number of the utilization of each medical and therapy service was assessed. Further, the number of days spent in inpatient treatment in a hospital and inpatient, or outpatient rehabilitation were surveyed.

#### Other relevant variables

The socio-demographic data were assessed during the baseline survey and included information about age and sex of the participants and current living situation of the patients (living alone or with a spouse/partner).

The participants were queried regarding their smoking habits and were classified as current, former, or never smokers. The Body-Mass-Index (BMI) was calculated by dividing the weight in kilograms by the height in squared meters^22^. Additionally, the participants provided information on previous strokes and important comorbidities: diabetes, coronary heart disease and cancer.

To assess the stroke severity and impairment after the stroke, the National Institutes of Health Stroke Scale (NIHSS) and the Modified Rankin Scale (mRS) were recorded at hospital discharge. For the NIHSS the patient is rated in 11 different subdomains: level of consciousness (LOC), LOC questions, LOC commands, gaze, visual fields facial palsy, motor arm, motor leg, ataxia, sensory, language, dysarthria, and extinction/inattention. Each subdomain is evaluated individually with a score ranging from 0 to 2, 3, or 4. The maximum overall score is 42, with higher scores indicating more sever neurological symptoms^23–25^. For the mRS the degree of impairment of the stroke patient is rated on a scale from 0 (no symptoms at all) to 6 (death)^26^.

The stroke impact scale (SIS) was part of the 3-month follow-up questionnaire^27^. We used the ‘activities of daily living’ (ADL/IADL) subscale to assess the patients’ limitations in everyday life at time of follow-up.

### Statistical methods

#### Statistical analysis

For description of the baseline characteristics, the metric-scaled PHQ-9 score was divided in 3 subgroups: “no depression” for scores under 5, “mild depression” for scores between 5 and 9 and “moderate to severe depression” for a score of 10 points and higher. All baseline variables were stratified for these 3 subgroups. Categorical variables are given as numbers (*n*) and percentages (%). Normal distributed variables are presented as mean ± standard deviation (*SD*). Median, 25th and 75th quartiles (*IQR*) are used to describe not-normally distributed variables.

Sample characteristics were given stratified by three subgroups, and differences were tested using the Pearson Chi^2^-Test or Fisher-Exact Test. The Kruskal-Wallis-Test was used for metric variables.

To determine the association between post-stroke depressiveness (assessed via PHQ-9 at baseline survey) and utilization of medical services, inpatient hospital stays, therapy services and rehabilitation at the 3-month follow-up binary logistic regression analyses were conducted. Therefore, the PHQ-9 score was used as a continuous variable. To gain deeper insights in the utilization of medical services an additional multivariable adjusted linear regression analysis was performed for the category “GP and/or internist”. Due to high missing values in the number of utilizations of other physicians, therapy services, days at hospital, days at inpatient rehabilitation and days at outpatient rehabilitation a multivariable adjusted linear regression analysis was not conducted for these outcomes.

#### Selection of covariables

A directed acyclic graph (DAG) was constructed based on a literature review to identifying causal and non-causal structures, confounders, and other types of bias^28^. Figure S1 (*Supplementary information*) shows the DAG of the direct effect of depressiveness, measured by the PHQ-9, at baseline survey on the utilization of healthcare services at the 3-month follow-up. Therefore, the web application “DAGitty” version 3.1 was used (free software under the GNU General Public License^29^.

According to the DAG, all regression models were adjusted for the variables sex, age, living situation, BMI, smoking behavior, comorbidities, former stroke events, stroke severity and impairment (measured with mRS and NIHSS).

#### Validation of assumptions for the regression analyses

The assumptions underlying the binary logistic regression analysis were validated. The independence of the observations is guaranteed by the study design. The presence of multicollinearity was evaluated by examining the variance inflation factor (VIF). Furthermore, the linearity assumption of continuous variables was tested using the Box-Tidwell procedure^30^. All variables exhibited a linear relationship in all binary logistic regression models.

The assumptions of the multivariable adjusted linear regression analysis were also tested. The linear relationship between the dependent and the independent variables was examined using a quantile-quantile plot and partial regression models for the metric variables. The calculated leverage values were screened for values exceeding 0.2^31^ and the Cook distance values were screened for values exceeding 1, indicating the presence of powerful outliers. The assumption of homoscedasticity was examined with scatterplots of the residuals and the Modified Breusch-Pagan Test. The results of the test indicated that the assumption of homoscedasticity was not sufficiently fulfilled. Consequently, the parameters of the model were calculated with the heteroskedasticity-consistent standard error HC3^32^. The residuals were examined for a normal distribution using a histogram and a P-P plot.

Outliers were not excluded from the binary logistic regression analyses or the multivariable adjusted linear regression analysis. It was assumed that these data were accurate and free from measuring errors. The exclusion of this data would have resulted in the biasing of the results.

#### Missing data

Observations with missing data for the PHQ-9 score at baseline or without 3-month follow-up were excluded. In the binary logistic regression analyses and the multivariable adjusted linear regression analysis, observations were excluded if at least one of the variables in the regression model was missing (complete case analysis).

#### Additional analyses and sensitivity analyses

In addition to the main analyses, a comparison of major characteristics between the patients included and excluded from the study was performed. Moreover, we calculated logistic regression analyses using the outcomes ‘psychiatrist/psychotherapist’ and ‘use of antidepressant medication at 3 month-follow-up’. Finally, we calculated the main regression models including only patients aged 50 years and older in order to test the association in a more homogenous study sample.

An alpha level of 0.05 was defined for all tests and 95% confidence intervals were provided. Statistical analysis was conducted using IBM^®^ SPSS^®^ Statistics, version 29.0.2.0 and the statistic software R version. 4.4.3.

### Further annotations

To improve the quality of the writing, the large language model “DeepL Write” was used^33^.

## Results

### Sample characteristics

#### Baseline characteristics

Table 1 represents the baseline characteristics for the total study sample and stratified for the three depression groups. The majority of patients included were men (59.0%) and the age of the participants ranged between 20 and 95 years. The mean age of the participants was 68.2 years, with a *SD* of 12.5 years. In total, 69.0% of the participants were living with their partner or spouse. The median BMI was 26.5 (*IQR*: 24.0-30.1). Additionally, 44.0% of the participants were former smokers. A total of 39.4% of the patients had comorbidities, and for 79.5% it was their first stroke event. The median value of the NIHSS at hospital discharge was 0 (*IQR*: 0.0–1.0) and 41.4% of the participants had a mRS score of 0.

Supplementary table S1 displays the characteristics of the patients excluded from the regression analyses. It reveals that the patients included into regression analyses were younger than patients with missing PHQ-9 information, but about the same age as patients without 3-month follow-up or with missing values for relevant covariables. The proportion of males was the highest in the group of patients included into regression analyses. Patients excluded from the analysis had the more severe events represented by higher NIHSS and mRS values. Moreover, there were significant differences between the groups regarding the living situation, smoking, activities of daily living (3-month follow-up) and use of antidepressant medication at 3-month follow-up.

#### Depressiveness at baseline

The PHQ-9 scores in the total sample ranged from 0 to 21 and the mean score was 4.8 (median: 4.0). According to the classification scheme, 312 participants (57.1%) exhibited no PSD, 156 patients (28.8%) were classified as having a mild depression and 78 (14.9%) as having a moderate to severe depression.

#### Utilization of healthcare services 3 months post-stroke

In total, 96.5% of the participants visited the GP and/or internist in the last 3 months, the median number of visits was 4 for patients with mild depression and 3 for patients with no depression and mild to severe depression (Table 2). Other physicians were utilized by 70.1% of the patients. The difference between subgroups was found to be statistically significant (*p* = 0.006). Therapy services were utilized by 48.2% of the participants. In total, 17.4% of the participants had inpatient treatment at the hospital with increasing proportion for more severe degrees of depressiveness. Furthermore, 55.2% of the patients did not utilize inpatient or outpatient rehabilitation with a statistically significant difference between the 3 subgroups (*p* = 0.002). Further details are presented in Table 2.

**Table 1:** Baseline characteristics of the sample for the total group and stratified for the 3 subgroups of depressiveness: no depression, mild depression and moderate to severe depression.

Variable	*n*	Total *n* = 546	Depressiveness at baseline according to PHQ-9^1^ score			
			No depression *n* = 312	Mild depression *n* = 156	Moderate–severe depression *n* = 78	*p*-value
Sex	546					
Male		322 (59.0)	201 (64.4)	83 (53.2)	38 (48.7)	**0.009**
Female		224 (41.0)	111 (35.6)	73 (46.8)	40 (51.3)	
Age in years (mean, SD)	546	68.2 (12.5)	68.8 (12.6)	68.1 (11.9)	66.3 (13.2)	0.292
Living situation	546					
Living with spouse/partner		377 (69.0)	217 (69.6)	108 (69.2)	52 (66.7)	0.884
Living alone		169 (31.0)	95 (30.4)	48 (30.8)	26 (33.3)	
BMI (median, IQR)	540	26.5 (24.0–30.1)	26.2 (23.9–30.0)	26.8 (24.2–30.2)	26.7 (24.2–30.1)	0.742
Smoking	546					
Current smoker		80 (14.7)	44 (14.1)	22 (14.1)	14 (17.9)	0.837
Former smoker		240 (44.0)	136 (43.6)	68 (43.6)	36 (46.2)	
Never smoker		226 (41.4)	132 (42.3)	66 (42.3)	28 (35.9)	
Comorbidities²	546					
Yes		215 (39.4)	118 (37.8)	64 (41.0)	33 (42.3)	0.679
No		331 (60.6)	194 (62.2)	92 (59.0)	45 (57.7)	
Etiology (TOAST)	495					
Large artery atherosclerosis		131 (26.5)	81 (28.0)	32 (24.1)	18 (24.7)	0.575
Cardioembolism		122 (24.6)	74 (25.6)	28 (21.1)	20 (27.4)	
Small vessel disease		102 (20.6)	52 (18.0)	35 (26.3)	15 (20.5)	
Other/undetermined		140 (28.3)	82 (28.4)	38 (28.6)	20 (27.4)	
Stroke type	546					
Ischemic		528 (96.7)	304 (97.4)	146 (93.6)	78 (100.0)	0.019
Hemorrhagic		18 (3.3)	8 (2.6)	10 (6.4)	0 (0.0)	
Former stroke	546					
Yes		112 (20.5)	65 (20.8)	32 (20.5)	15 (19.2)	0.952
No		434 (79.5)	247 (79.2)	124 (79.5)	63 (80.8)	
NIHSS^3^ at hospital discharge (median, IQR)	478	0.0 (0.0–1.0)	0.0 (0.0–1.0)	0.0 (0.0–1.0)	0.0 (0.0–1.0)	0.740
NIHSS^3^ at hospital discharge (mean, SD)		0.9 (1.5)	0.9 (1.5)	0.9 (1.4)	0.8 (1.5)	0.850
mRS^4^ at hospital discharge	478					
mRS score 0		221 (41.4)	129 (42.0)	61 (40.1)	31 (41.3)	0.116
mRS score 1		140 (26.2)	84 (27.4)	42 (27.6)	14 (18.7)	
mRS score 2		101 (18.9)	58 (18.9)	23 (15.1)	20 (26.7)	
mRS score 3		47 (8.8)	21 (6.8)	21 (13.8)	5 (6.7)	
mRS score 4–5		25 (4.7)	15 (4.9)	5 (3.3)	5 (6.7)	
Activities of daily living^5^ at 3-month-FU (median, IQR)	532	95.8 (83.3–100.0)	95.8 (87.5–100.0)	93.8 (83.3–97.9)	89.6 (75.0–95.8)	**< 0.001**
Antidepressant medication at 3-month-FU	546	55 (10.1)	21 (6.7)	16 (10.3)	18 (23.1)	**< 0.001**

^1^Patient Health Questionnaire-9: the participant rates 9 questions, in total a score of maximum 27 can be reached where a higher score indicates a higher degree of depressiveness.

²Diabetes, coronary heart disease and/or cancer.

^3^National Institutes of Health Stroke Scale: the maximum total score is 42. Higher scores indicate greater severity of neurological symptoms.

^4^Modified Rankin Scale: a score of 0 indicates the absence of symptoms, a score of 1 indicates no significant disability, a score of 2 indicates a slight disability, a score of 3 indicates a moderate disability, a score of 4 indicates a moderately severe disability and a score of 5 indicates a severe disability.

^5^ADL/IADL subscale of the stroke impact scale (SIS).

Significant values are in bold.

**Table 2:** Descriptive analysis of the utilization of healthcare services of the sample for the total group and stratified for the 3 subgroups of depressiveness: no depression, mild depression and moderate to severe depression.

Variable	n^2^	Total	Depressiveness at baseline according to PHQ-9^1^ score			
			No depression *n* = 409	Mild depression *n* = 222	Moderate–severe depression *n* = 123	p-value
Physicians						
GP^3^/internist	536					
No		19 (3.5)^4^	12 (3.9)	5 (3.3)	2 (2.6)	0.948^a^
Yes		517 (96.5)	294 (96.1)	147 (96.7)	76 (97.4)	
Number, median (IQR^5^*)*	490	3.0 (3.0)	3.00 (3.0)	4.0 (3.0)	3.0 (3.5)	0.086^b^
Other physicians	441					
No		132 (29.9)	89 (36.0)	28 (21.1)	15 (24.6)	**0.006** ^c^
Yes		309 (70.1)	158 (64.0)	105 (79.0)	46 (75.4)	
Therapy services	512					
No		265 (51.8)	161 (55.0)	71 (49.0)	33 (44.6)	0.206^c^
Yes		247 (48.2)	132 (45.1)	74 (51.0)	41 (55.4)	
Treatment at hospital	535					
No		442 (82.6)	262 (84.5)	119 (80.4)	61 (79.2)	0.392^c^
Yes		93 (17.4)	48 (15.5)	29 (19.6)	16 (20.8)	
Rehabilitation	527					
No		291 (55.2)	186 (61.6)	67 (44.7)	38 (50.7)	**0.002** ^c^
Yes		236 (44.8)	116 (38.4)	83 (55.3)	37 (49.3)	

Significant values are in bold.

^1^Patient Health Questionnaire-9: the participant rates 9 questions, in total a score of maximum 27 can be reached where a higher score indicates a higher degree of depressiveness.

^2^Number of participants.

^3^General practitioner.

^4^Values are expressed as numbers (percentage) unless otherwise indicated.

^5^Interquartile range.

^a^Fisher–Exact Test.

^b^Kruskal–Wallis-Test.

^c^Pearson Chi^2^-Test.

### Association between depressiveness and utilization of healthcare services

The self-reported depressiveness significantly influenced the frequency of utilization of GP and/or internists as well as the utilization of inpatient treatments at hospital and of inpatient or outpatient rehabilitation. The results of the binary logistic and the multivariable adjusted linear regression analyses are presented in Table 3 and in the following sections.

An OR greater than 1 indicates that patients in the given group were more likely to utilize the respective healthcare discipline; an OR less than 1 indicates that participants in the respective group were less likely to utilize the respective healthcare discipline. A positive beta estimate indicates the average increase in the frequency of utilization of other physicians increases when the independent variable (PHQ-9 score) increases by one point.

**Table 3:** Results of the binary logistic and the multivariable adjusted linear regression analyses for the utilization of the respective healthcare services.

	Utilization of GPs^1^ and/or internists (yes/no)^a^ *n* = 464			Frequency of utilization of GPs and/or internists^b^ *n* = 427		
	OR2	95% CI^3^	*p*-value	Beta	95% CI	*p*-value
PHQ-9 score^4^	1.06	0.92–1.23	0.434	0.10	0.01–0.19	**0.036**

	Utilization of other physicians (yes/no)^a^ *n* = 385			Utilization of therapy services (yes/no)^a^ *n* = 443		
	OR	95% CI	*p*-value	OR	95% CI	*p*-value
PHQ-9 score	1.06	1.00–1.12	0.067	1.04	0.99–1.10	0.103

	Utilization of inpatient hospital treatment (yes/no)^a^ *n* = 463			Utilization of inpatient or outpatient rehabilitation (yes/no)^a^ *n* = 454		
	OR	95% CI	*p*-value	OR	95% CI	*p*-value
PHQ-9 score	1.07	1.01–1.14	**0.016**	1.09	1.03–1.15	**0.003**

Models adjusted for: sex, age, living situation, Body Mass Index, smoking behavior, comorbidities, former stroke events, stroke severity and impairment.

Significant values are in bold.

^a^Binary logistic regression analysis.

^b^Multivariable adjust linear regression analysis with heteroskedasticity-consistent standard error HC3.

^1^General practitioner.

^2^Odds ratio.

^3^95% Confidence interval.

^4^Patient Health Questionnaire-9: the participant rates 9 questions, in total a score of maximum 27 can be reached where a higher score indicates a higher degree of depressiveness.

As shown in Table 3, the binary logistic regression analysis demonstrated a positive but not statistically significant association of the depressiveness at baseline and the utilization of GPs and/or internists (OR = 1.06; 95% CI: 0.92–1.23; *p* = 0.434).

In contrast, the multivariable adjusted linear regression model did show a significant positive association between the depressiveness at baseline and the frequency of visits of GPs and/or internists (β = 0.10; 95% CI: 0.01–0.19; *p* = 0.036), indicating that patients with higher PHQ-9 scores utilized GPs and/or internists more often.

The results of the binary logistic regression analysis demonstrated a positive but not statistically significant association of the depressiveness at baseline and the utilization of other physicians (OR = 1.06; 95% CI: 0.996–1.120; *p* = 0.067), indicating that patients with higher PHQ-9 scores were more likely to utilize other physicians (Table 3).

Also, a positive but not statistically significant association of the depressiveness at baseline and the utilization of therapy services (OR = 1.04; 95% CI: 0.99–1.10; *p* = 0.103) could be demonstrated, indicating that patients with higher PHQ-scores were more likely to utilize therapy services.

A positive and statistically significant association of the depressiveness at baseline and the inpatient treatment at the hospital (OR = 1.07; 95% CI: 1.01–1.14; *p* = 0.016) was found, indicating that patients with higher PHQ-9 scores were more likely to have an inpatient treatment at the hospital.

Furthermore, the observed positive and statistically significant association of the depressiveness at baseline and the utilization of inpatient or outpatient rehabilitation (OR = 1.09; 95% CI: 1.03–1.15; *p* = 0.003), indicated that patients with higher PHQ-scores were more likely to utilize inpatient or outpatient rehabilitation.

### Supplementary results

The sensitivity analyses including only patients aged 50 years and older revealed very similar association between PHQ-9 and utilization of health care services, see table S2 in the supplementary material.

There was no significant association between PHQ-9 and visiting a psychiatrist/psychotherapist, see supplementary table S3. However, there was a significant positive association between PHQ-9 and use of antidepressant medication an 3 month-follow up (OR: 1.11 [1.05–1.17], p value: <0.001).

## Discussion

### Major findings

The results of the present study revealed that nearly half of the patients experienced a degree of depressiveness in the acute phase after stroke. Furthermore, the results demonstrated a positive association between self-reported depressive symptoms and healthcare utilization 3 months after the stroke event across all categories. This suggests that as depressiveness increases, the utilization of healthcare services rises. However, statistical significance was only observed for the number of visits at GPs and/or internists, inpatient hospital treatment and inpatient or outpatient rehabilitation.

A recent population-based study from Sweden reported that individuals with (treatment-resistant) depression had an increased utilization of health care resources^34^. Another study found that patients with depressive symptoms but without clinically diagnosed depression also had a higher utilization of health care services compared to individuals without depressive symptoms(e.g. GP visits)^35^. Also in multimorbid elderly patients, a study by Bock et al. reported that depression was associated with increased health care utilization and costs^36^. These results are confirmed by a review from 2012 which came to the conclusion that there is a positive association between depressive symptoms and health service utilization in late life^37^. All these studies and results are majorly in line with the findings of the present study. Nevertheless, to this date and to the best of our knowledge, not much research has been conducted that analyzed the association between symptoms of depression and utilization of healthcare services specifically in stroke patients, so comparison of our results with existing literature is quite challenging. However, in a study on 366 patients with myocardial infarction, Schlyter et al. reported that depression did not predict increased health care utilization^38^. These discrepancies might be due to the different underlying diseases (myocardial infarction vs. stroke) but could be also caused by methodological differences between the different studies.

In the present study, the incidence of PSD (defined as a PHQ-9 value of greater than 4^18^ ) was present in 45.8% o the included patients. This is higher than the incidence of one-third of stroke survivors that is reported in the literature^8,9^. One potential explanation for this discrepancy is that in the present study, the depression was self-reported by the patients, whereas in the literature confirmed depression diagnoses are reported.

### PSD and utilization of gps and/or internists

In the multivariable adjusted linear regression analysis, a statistically significant association between self-reported depressiveness and utilization of GPs and/or internists 3 months after stroke was found. One potential explanation for this finding is the gatekeeper function of GPs. A study conducted by the National Association of Statutory Health Insurance Physicians revealed that in 2021, 80% of individuals insured through a statutory health insurance visited a physician, with 81% of these individuals visiting a GP^39^. Our results confirm that GPs are broadly utilized also by post-stroke patients, in particular more frequently by patients with PSD.

### PSD and inpatient hospital stay

Two previous studies conducted in the Anglo-American region examined the healthcare utilization of veterans after a stroke. Both studies demonstrated that veterans diagnosed with a PSD had significantly more inpatient hospitalization, prolonged inpatient hospitalization stays, and augmented outpatient utilization compared to veterans without a mental health diagnosis^40,41^. A further study of patients who had undergone inpatient stroke rehabilitation revealed that patients with 2 or more mental health conditions, such as depression or anxiety, had a significantly elevated risk of mortality or rehospitalization^42^.

The results of these 3 studies, which focused on the hospitalization of patients, are confirmed by the findings of the present analysis, which indicated that a higher PHQ-9 score was significantly associated with a higher likelihood of inpatient treatment at a hospital. One potential explanation for this phenomenon is the association of depression with several adverse outcomes, including lower quality of life, mortality and disability^12^. Such adverse outcomes may result in an increased requirement for inpatient hospital treatment.

### PSD and utilization of other physicians and therapy services

The present study showed a positive but not significant association between the utilization of other physicians and therapy services and PSD. There are only a few prior studies that have evaluated the association between PSD and healthcare utilization and, to the best of our knowledge, no studies specifically examining the utilization of medical specialists and therapy services. This lack of knowledge was also pointed out by Towfighi et al. and therefore they recommended further studies to evaluate this topic^10^. Consequently, the question of why the present study did not show a significant association between the utilization of other physicians and therapy services and PSD must remain unanswered.

### Survey instruments and questionnaires

In the SCHANA study the overarching subject of stroke was approached from various perspectives using standardized assessments wherever feasible. Thus, the mRS and the NIHSS were utilized to screen for impairment and stroke severity. The NIHSS is one of the most commonly employed assessments for rating deficits subsequent to a stroke, particularly in clinical trials^14–16^. The mRS was developed for the purpose of assessing global disability and recovery from stroke^43^. It is a highly valid and reliable assessment and thus, it is frequently employed in randomized controlled trials.

Furthermore, the PHQ-9 questionnaire was used to assess the depressiveness of the participants. It is important to note that the PHQ-9 is not a substitute for a clinical diagnosis of depression^19^. Nevertheless, the PHQ-9 questionnaire is a screening tool for distinguishing between patients with and without post-stroke depressive disorders^44^. The PHQ-9 questionnaire was selected for the present study due to its brevity and simple handling to assess the patient-reported depressiveness^21^.

The PHQ-9 measures symptoms of depression, which must be differentiated from post-stroke apathy. The latter is characterized by loss of motivation and goal directed activity after stroke^45,46^. It can deviate from actual depressiveness in terms of symptomatology and post-stroke apathy might tend to persist longer after the acute event^46^. Like post-stroke depression, post-stroke apathy is also associated with poorer outcome and worse prognosis after stroke^45^. The impact of antidepressants on post-stroke apathy however is rather unclear at the moment^46^.

### Strength and limitations

The present study’s prospective design, with a follow-up survey 3 months after stroke, is one of its most significant strengths. The study was conducted at one of the largest stroke centers in Germany, with approximately 2,000 stroke patients treated annually^47^.

However, one major limitation of the SCHANA study is that the outcome of the present analysis is entirely based on patient self-report. This must be taken into account when interpreting the results, as this might cause a recall bias^48^. A previous study demonstrated that self-reported healthcare utilization tends to be underreported, which may lead to biased information in the present study^49^. Furthermore, the data regarding the patient’s pre-stroke diagnosis of depression is also based on information provided by the patient. In addition, the utilization of postal questionnaires or telephone interviews must be subjected to critical analysis. The target group of stroke patients is predominantly composed of older individuals, with the median age at the time of the first stroke being 73 years in Europe^50^. Furthermore, the participants may have experienced minor to major impairments as a result of the stroke event, which could influence their ability to understand and respond to the questionnaire. The results of the missing value analysis of the present study, which demonstrated that patients excluded from the regression analyses were significantly older, had higher NIHSS- and mRS-scores, and were more likely to live alone, confirmed this theory. In the present study, this resulted in a high number of missing values, which reduced the statistical power of the study and potentially introduced a selection bias into the results, as patients with more severe impairments exhibited significantly higher rates of missing values.

Nevertheless, the decision to utilize postal questionnaires for the follow-up survey is also associated with advantages. In particular for patients with disabilities resulting from stroke, responding to a postal questionnaire may be a more convenient alternative to arranging a transport to a study center. Furthermore, questionnaires can help to minimize social-desirability bias.

The timing of the conduction of the PHQ-9 questionnaire must be evaluated critically. In the present study, the assessment was conducted at the time of discharge from the hospital, which is in Germany typically 8 to 10 days after the stroke event^51^. As PSD can manifest at any point after stroke^10^, the generalizability of the findings of this study to patients with a later-onset PSD may be limited. In-hospital the patient may not be aware of the extent and consequences of the stroke which only become evident after discharge e.g. when the patient is confronted with challenges in their home environment. Consequently, screening at a later time point might have been preferable in some regard. However, the majority of patients will experience symptoms of PSD within the first 3 months after a stroke^52^. As demonstrated by Liu et al., especially early-onset PSD can have severe consequences for the patients, including an increased risk of subsequent stroke events^13^. Therefore, the conduction of the PHQ-9 assessment at the time of hospital discharge can be considered an appropriate method for identifying early-onset PSD in our view.

Another limitation of the study is the lack of information on post-stroke apathy, so we were not able to differentiate between depressive symptoms and post-stroke apathy.

The external validity and the generalizability of the results of SCHANA study are limited, as the study population is comprised solely of patients treated at the University Hospital Augsburg and severe stroke cases are underrepresented. It must be taken into account that the inhabitants of the Augsburg region might not be representative of the entire German population with regards to socio-demographic factors such as age, education and previous illnesses. Therefore, the results must be interpreted with caution, when attempting to generalize them to other regions.

### Implications for further research

As there is a lack of available data regarding the utilization of healthcare services in patients with a PSD, particularly in Germany, the present study offers an important initial insight into this topic. The present study examined the association between depressive symptoms with an early onset and the utilization of healthcare services in the first 3 months after the stroke event. While the need for medical services and the costs are highest in this period^53^, it is also important to examine this association in further research over a longer period of time to evaluate the long-term effects.

Further analyses could be conducted on the basis of health insurance data in order to eliminate a recall bias and to obtain information regarding the utilization of medical services among patients with confirmed clinical diagnoses of depression. Furthermore, an analysis of health insurance data allows an evaluation of the quality of the treatment of a PSD. A study in the US demonstrated, that two-thirds of the patients that were diagnosed with a PSD did not receive any treatment therefore^14^. With regard to the various potential adverse consequences of PSD^9–13^, it is imperative to conduct an investigation of the current treatment landscape, such as the use of digital therapy services, which represent an opportunity to mitigate the burden on the healthcare system.

## Conclusion

The present study provided insights into the association between PSD and the healthcare utilization in Germany from a patient’s perspective. The findings suggested an association between elevated depressiveness scores and an increased utilization of healthcare services. This highlights the importance of a consequent screening process for PSD and subsequent treatment, as an existing PSD is a major risk factor for a negative post-stroke outcome. In the context of the rising incidence of stroke attributed to the demographic shifts and the strain on healthcare systems, this is of high importance, given the more intensive utilization of healthcare services by patients with post-stroke depressiveness. Further studies are required to gain a more comprehensive understanding of the association between PSD and healthcare utilization.

## Supplementary Information

Below is the link to the electronic supplementary material.

Supplementary Material 1

## Data Availability

The raw data supporting the conclusions of this article will be made available by the authors upon reasonable request. Please contact the corresponding author Timo Schmitz (mail: timo.schmitz@med.uni-augsburg.de).
