# Supplementary material for: Association between post-stroke depressiveness and the utilization of healthcare services three months after the stroke
Source: Sci Rep. 2025 Aug 2;15:28281. doi: 10.1038/s41598-025-12875-x (PMC12318013; doi:10.1038/s41598-025-12875-x)
Supplement: Supplementary file 1 — Supplementary Material 1 [file 41598_2025_12875_MOESM1_ESM.pdf]

Supplementary material

Title: Association between post-stroke depressiveness and the utilization of healthcare services three months after the stroke event

Raphaela Artner, Christine Meisinger, Michael Ertl, Markus Naumann, Jakob Linseisen*, Timo Schmitz*
*shared last authorship

Table S1: Baseline characteristics of patients excluded due to missing values for the PHQ-9 score, patients excluded without 3-month follow-up, patients excluded due to missing values for relevant covariables and patients included into regression analyses.

**Variable**	**Total sample** *n*=945	**Missing PHQ-9** *n*=191	**No 3-month follow-up** *n=208*	**Missing data on relevant covariables** *n=73*	***Cases included into regression analyses*** *n=473*	***p*-value**
Sex						
Male	531 (56.2)	91 (47.6)	118 (56.7)	39 (53.4)	283 (59.8)	**0.037**
Female	414 (43.8)	100 (52.4)	90 (43.3)	34 (46.6)	190 (40.2)	
Age in years (*mean, SD)*	69.4 (13.1)	73.7 (12.4)	68.5 (14.4)	67.4 (12.8)	68.4 (12.4)	**<0.001**
Living situation						
Living with spouse/partner	494 (64.7)	5 (55.6)	112 (53.8)	47 (64.4)	330 (69.8)	**0.001**
Living alone	269 (35.3)	4 (44.4)	96 (46.2)	26 (35.6)	143 (30.2)	
BMI (median, *IQR)*	26.3 (23.8 - 29.7)	26.1 (23.2 - 29.3)	25.9 (23.4 - 29.1)	27.0 (24.2 - 29.4)	26.4 (23.9 - 30.2)	0.387
Smoking						
Current smoker	125 (13.2)	2 (1.0)	43 (20.7)	11 (15.1)	69 (14.6)	**<0.001**
Former smoker	344 (36.4)	13 (6.8)	91 (43.8)	35 (47.9)	205 (43.3)	
Never smoker	476 (50.4)	176 (92.1)	74 (35.6)	27 (37.0)	199 (42.1)	
Comorbidities						
Yes	310 (39.5)	13 (43.3)	82 (39.4)	32 (43.8)	183 (38.7)	0.828
No	474 (60.5)	17 (56.7)	126 (60.6)	41 (56.2)	290 (61.3)	
Etiology (TOAST)						
large artery atherosclerosis	223 (25.5)	45 (24.9)	47 (23.9)	22 (33.3)	109 (25.4)	0.169
cardioembolism	219 (25.1)	46 (25.4)	51 (25.9)	22 (33.3)	100 (23.3)	
small vessel disease	163 (18.7)	29 (16.0)	32 (16.2)	8 (12.1)	94 (21.9)	

other/undetermined	268 (30.7)	61 (33.7)	67 (34.0)	14 (21.2)	126 (29.4)	
Stroke type						
ischemic	908 (96.1)	183 (95.8)	197 (94.7)	70 (95.9)	458 (96.8)	0.618
hemorrhagic	37 (3.9)	8 (4.2)	11 (5.3)	3 (4.1)	15 (3.2)	
Former stroke						
Yes	625 (79.6)	22 (71.0)	169 (81.2)	58 (79.5)	376 (79.5)	0.620
No	160 (20.4)	9 (29.0)	39 (18.8)	15 (20.5)	97 (20.5)	
NIHSS at hospital discharge (median, *IQR)*	0.0 (0.0 - 2.0)	1.0 (0.0 - 2.0)	1.0 (0.0 - 2.0)	1.0 (0.0 - 2.0)	0.0 (0.0 - 1.0)	**<0.001**
NIHSS at hospital discharge (mean, *SD)*	1.3 (2.2)	1.9 (3.2)	1.7 (2.4)	1.6 (2.1)	0.9 (1.5)	**<0.001**
mRS at hospital discharge						
mRS score 0	352 (37.8)	60 (31.9)	71 (34.1)	4 (6.6)	217 (45.9)	**<0.001**
mRS score 1	219 (23.5)	40 (21.3)	39 (18.8)	20 (32.8)	120 (25.4)	
mRS score 2	156 (16.8)	24 (12.8)	31 (14.9)	21 (34.4)	80 (16.9)	
mRS score 3	109 (11.7)	27 (14.4)	35 (16.8)	11 (18.0)	36 (7.6)	
mRS score 4-5	94 (10.1)	37 (19.7)	32 (15.4)	5 (8.2)	20 (4.2)	
activities of daily living 5 at ***3-month-FU*** (median, *IQR)*	93.8 (81.2 - 100.0)	79.2 (27.1 - 95.0)	not available	91.7 (79.2 - 97.9)	95.8 (83.3 - 100.0)	**<0.001**
Antidepressant medication at ***3-month-FU***	124 (13.1)	41 (21.5)	not available	6 (8.2)	49 (10.4)	**0.001**

Table S2: Results of the regression analyses including only patients aged 50 years and older.

	**Utilization of GPs[1] and/or internists[a]**			**Frequency of utilization of GPs and/or internists[b]**		
	OR	95 % CI[4]	*p*-value	Beta	95 % CI	*p*-value
**PHQ-9 score[5]**	1.06	0.93 – 1.25	0.447	0.09	0.00 - 0.19	0.068
	**Utilization of other physicians[a]**			**Utilization of therapy services[a]**		
	OR	95 % CI	*p*-value	OR	95 % CI	*p*-value
**PHQ-9 score**	1.06	0.99 – 1.12	0.081	1.05	0.99 – 1.11	0.087
	**Utilization of inpatient hospital treatment[a]**			**Utilization of inpatient or outpatient rehabilitation[a]**		
	OR	95 % CI	*p*-value	OR	95 % CI	*p*-value
**PHQ-9 score**	1.07	1.01 – 1.14	**0.026**	1.11	1.05 – 1.18	**0.001**

[a] Binary logistic regression analysis
[b] Multivariable adjusted linear regression analysis with heteroskedasticity-consistent standard error HC3
[1] General practitioner

Table S3: Results of the logistic regression analyses for the outcomes utilization of psychiatrist/psychotherapist and use of antidepressant medication at 3-month follow-up.

	**Utilization of psychiatrist/psychotherapist**			**Use of antidepressant medication**		
	OR	95 % CI	*p*-value	OR	95 % CI	*p*-value
**PHQ-9 score**	1.08	0.97 – 1.19	0.173	1.11	1.05 – 1.17	**<0.001**

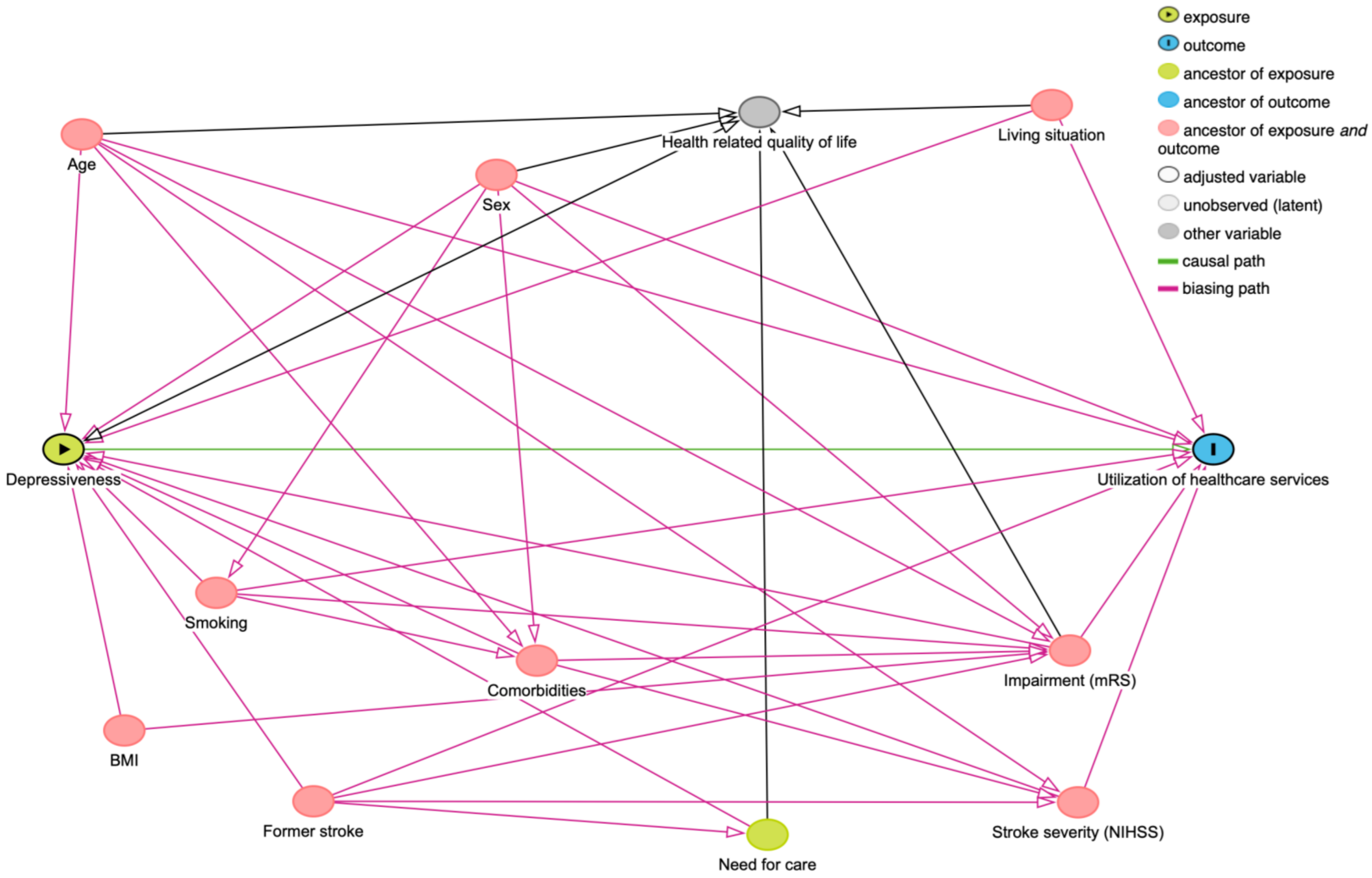

Figure S1. DAG of the direct effect of depressiveness, measured by the PHQ-9, at baseline survey on the utilization of healthcare services at the 3-month follow-up with covariables
